# Current progress and future opportunities in applications of bioinformatics for biodefense and pathogen detection: report from the Winter Mid-Atlantic Microbiome Meet-up, College Park, MD, January 10, 2018

**DOI:** 10.1186/s40168-018-0582-5

**Published:** 2018-11-05

**Authors:** Jacquelyn S. Meisel, Daniel J. Nasko, Brian Brubach, Victoria Cepeda-Espinoza, Jessica Chopyk, Héctor Corrada-Bravo, Marcus Fedarko, Jay Ghurye, Kiran Javkar, Nathan D. Olson, Nidhi Shah, Sarah M. Allard, Adam L. Bazinet, Nicholas H. Bergman, Alexis Brown, J. Gregory Caporaso, Sean Conlan, Jocelyne DiRuggiero, Samuel P. Forry, Nur A. Hasan, Jason Kralj, Paul M. Luethy, Donald K. Milton, Brian D. Ondov, Sarah Preheim, Shashikala Ratnayake, Stephanie M. Rogers, M. J. Rosovitz, Eric G. Sakowski, Nils Oliver Schliebs, Daniel D. Sommer, Krista L. Ternus, Gherman Uritskiy, Sean X. Zhang, Mihai Pop, Todd J. Treangen

**Affiliations:** 1 Center for Bioinformatics and Computational Biology, University of Maryland, College Park, College Park, MD USA; 2 School of Public Health, University of Maryland, College Park, College Park, MD USA; 3 Material Measurement Laboratory, National Institute of Standards and Technology, Gaithersburg, MD USA; 4 National Biodefense Analysis and Countermeasures Center, Frederick, MD USA; 5 Bloomberg School of Public Health, Johns Hopkins University, Baltimore, MD USA; 6 The Pathogen and Microbiome Institute, Northern Arizona University, Flagstaff, AZ USA; 7 National Human Genome Research Institute, National Institutes of Health, Bethesda, MD USA; 8 Department of Biology, Johns Hopkins University, Baltimore, MD USA; 9 CosmosID, Inc., Rockville, MD USA; 10 Department of Pathology, University of Maryland School of Medicine, Baltimore, MD USA; 11 Maryland Institute for Applied Environmental Health, School of Public Health, University of Maryland, College Park, College Park, MD USA; 12 Environmental Health and Engineering, Johns Hopkins University, Baltimore, MD USA; 13 B.Next, In-Q-Tel, Inc., Arlington, VA USA; 14 Department of Computer Science, University of Tübingen, Tübingen, Germany; 15 Signature Science, LLC, Arlington, VA USA; 16 Division of Medical Microbiology, Department of Pathology, School of Medicine, Johns Hopkins University, Baltimore, MD USA; 17 Present address: Department of Computer Science – MS-132, Rice University, P.O. Box 1892, Houston, TX 77005-1892 USA

**Keywords:** Microbiome, Metagenomics, Bioinformatics, Biodefense, Biothreats, Pathogen detection, Longitudinal analysis

## Abstract

The Mid-Atlantic Microbiome Meet-up (M^3^) organization brings together academic, government, and industry groups to share ideas and develop best practices for microbiome research. In January of 2018, M^3^ held its fourth meeting, which focused on recent advances in biodefense, specifically those relating to infectious disease, and the use of metagenomic methods for pathogen detection. Presentations highlighted the utility of next-generation sequencing technologies for identifying and tracking microbial community members across space and time. However, they also stressed the current limitations of genomic approaches for biodefense, including insufficient sensitivity to detect low-abundance pathogens and the inability to quantify viable organisms. Participants discussed ways in which the community can improve software usability and shared new computational tools for metagenomic processing, assembly, annotation, and visualization. Looking to the future, they identified the need for better bioinformatics toolkits for longitudinal analyses, improved sample processing approaches for characterizing viruses and fungi, and more consistent maintenance of database resources. Finally, they addressed the necessity of improving data standards to incentivize data sharing. Here, we summarize the presentations and discussions from the meeting, identifying the areas where microbiome analyses have improved our ability to detect and manage biological threats and infectious disease, as well as gaps of knowledge in the field that require future funding and focus.

**Electronic supplementary material:**

The online version of this article (10.1186/s40168-018-0582-5) contains supplementary material, which is available to authorized users.

## Introduction

Strong public health and biodefense research is essential for the prevention, detection, and management of biological threats and infectious disease. Over the last century, the focus of biodefense research has shifted in response to modern advances in biotechnology. Specifically, a biological revolution is underway, generating promising new gene editing and synthetic biology technologies that may transform modern medicine, but also present a threat to public health if misappropriated [1]. As biotechnology becomes increasingly globalized, it is important that we establish new strategies and tools for infectious disease detection and surveillance that will help us protect against bioterrorism and manage disease outbreaks.

Rapid advances in next-generation sequencing (NGS) technologies have helped advance biodefense research by enabling the development of new methods for identifying and characterizing pathogens. Amplification and sequencing of the 16S rRNA gene allow for high-throughput detection of prokaryotic communities, while shotgun metagenomic sequencing approaches capture the composition and functional potential of multi-domain populations. Metagenomic analyses used for pathogen detection and identification are often time sensitive. The results help inform high-stakes decision-making, such as choosing an appropriate medical treatment, deciding if a food product should be recalled due to contamination, or determining if an area should be shut down due to a suspected act of bioterrorism. In addition, geospatial and temporal metagenomic analyses are essential for tracking the dynamic responses of microbial populations to changes in environmental or human health. However, improvements in precision, sensitivity, speed, cost, and accuracy of NGS and downstream analyses are necessary for effective utilization in biodefense research [2–6].

**Fig. 1:**
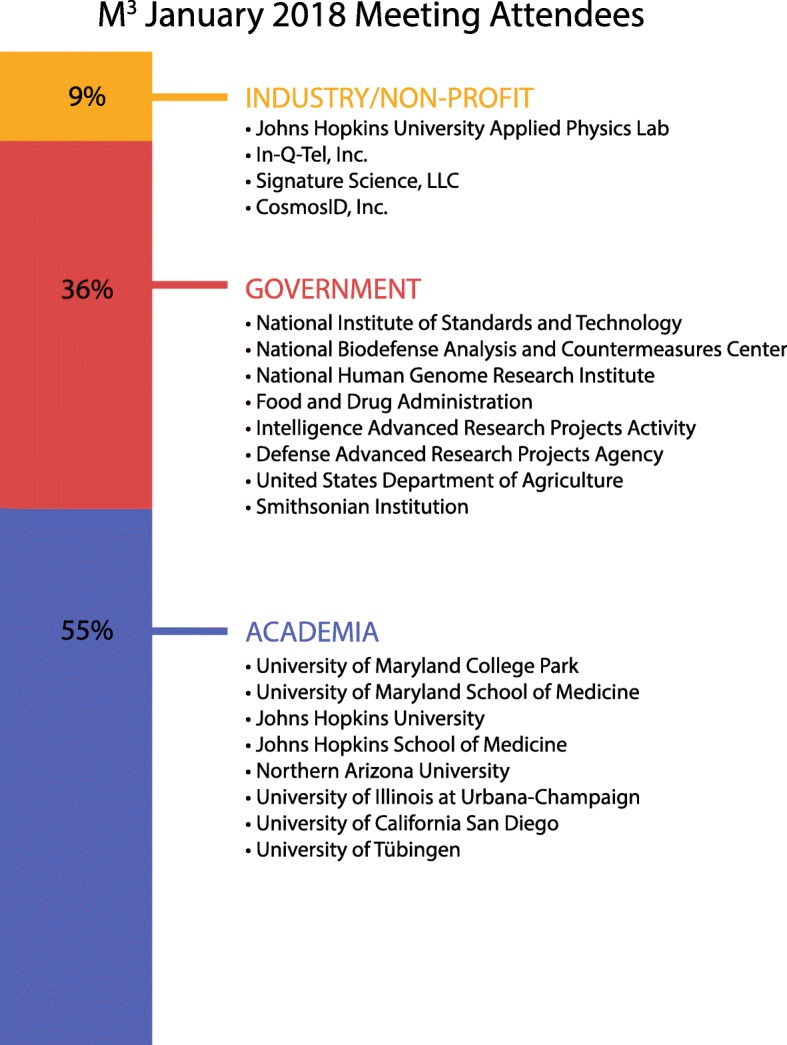
Different sectors and institutions represented at the January 2018 M^3^ Meet-up

On January 10, 2018, the Mid-Atlantic Microbiome Meet-up (M^3^) organization held a conference aimed at understanding how the biodefense and pathogen detection fields are transformed by new biological and computational technologies. While biodefense was broadly discussed, the participants focused primarily on emerging infectious disease applications. The meeting took place in the STAMP Student Union at the University of Maryland campus in College Park. The M^3^ consortium brings together microbiome researchers from different sectors to discuss challenges, develop standards and best practices, and help connect data generators with data analysts [7]. The M^3^ community is constantly growing and, as of this publication, has 140 members from over 25 different institutions. The conference was attended by 67 participants from academia, government, and industry (Fig. 1), with expertise in areas such as biodefense, computer science, genomics, microbiology, and public health. There were two talks given by invited speakers, 15 oral presentations selected from submitted abstracts, and several posters displayed at the meeting (Additional file 1: Table S1) [8]. Additionally, there were three interactive breakout sessions to address the challenges of the field and encourage networking (Additional file 1: Table S2). The event was sponsored in part by CosmosID, Inc., but they did not participate in the organization of the event nor in the selection of speakers and topics being discussed.

The tone for the meeting was set by the keynote address presented by Dr. Tara O’Toole, Executive Vice President of the non-profit strategic investor In-Q-Tel, Inc. Pointing to the problems in detection, containment, and treatment during the recent H1N9 pandemic and Ebola epidemic, Dr. O’Toole shared that current progress in the field is disappointing because biodefense is not a priority for any single government agency, funding support is irregular, and epidemics are becoming more common. Increasing international competition for biotechnology advancements and leadership make it even more important to stimulate progress.

Dr. O’Toole outlined several keys to innovation and policy, which were echoed by the presentations and discussions throughout the remainder of the meeting, including (1) the willingness to think anew, (2) development of new tools and instruments, (3) implementation of a technology-focused biodefense strategy, (4) delivery of near real-time situational awareness for existing epidemics by leveraging modern data analytics and networked communications, and (5) establishment of rich human networks and cross-sector partnerships between government agencies, the private sector, and academia.

## Key conclusions

We start by highlighting the key conclusions and recommendations identified by the participants in the meeting:

1. Sequencing-based assays frequently face challenges related to limits of detection and technical biases, and culturing or other enrichment strategies remain necessary in many applications. The accurate quantification of viable organisms or metabolic activity within complex metagenomic samples remains an open challenge that is unlikely to be solved through sequencing alone.
2. Current sample processing approaches tend to exclude viral and fungal/eukaryotic components of microbial communities. In the case of viruses, this problem is compounded by poor taxonomies and database resources.
3. Analytical approaches, community standards, and software for temporal data analysis have lagged behind the rapidly increased generation of such data.
4. Robust bioinformatics tools are critical for future progress. These tools must be developed to better match the needs of end users and must be subject to critical validation.
5. Data standards are essential for ensuring the quality and usefulness of shared datasets, but overly onerous reporting requirements discourage sharing. In cases where privacy is a concern, we must also develop solutions that allow for secure storage and processing of sensitive data.

These key recommendations are summarized in Table 1 and more extensively discussed below.

**Table 1:** Outline of current research gaps and future goals discussed at the January 2018 M^3^ Meeting

Research gaps	Current limitations	Community goals
Tracking microbial communities across time and topography (Key Conclusions 1 and 3) Importance: studies incorporating temporal and/or spatial sampling allow us to detect important shifts in community dynamics Application example: detecting the spread of infection in a hospital or of a pathogen contaminating crops and spreading food-borne illness	• Sequencing strategies are not able to quantify viable organisms (which is essential for biodefense applications) • Lack of well-established statistical approaches for exploring longitudinal microbiome data • Increased sample size makes these studies more expensive and harder to obtain sufficient statistical power for all subjects/time points/regions	• Collection, sequencing, and sharing of more time series datasets • Development of statistical methods and tools to help analyze longitudinal and/or geospatial microbiome datasets
Looking beyond bacterial pathogens (Key Conclusion 2) Importance: viral and fungal components of the microbiome are often under-explored, despite their potential implications in biodefense Application example: better understanding the transmission of infectious viruses, like influenza	• Lack of a universally distributed marker gene (viruses) • Difficult to obtain sufficient material from low biomass environments • High levels of host contamination • Incomplete databases	• More consistent database curation and maintenance (potentially incentivized financially or with publications) • Improved gene function identification
Development and application of metagenomic analysis tools (Key Conclusion 4) Importance: computational tools need to be developed to help improve the utility of high-throughput sequencing strategies for biodefense problems Application example: improved metagenome assembly methods could better delineate between different strains of a pathogen in samples	• Tools for metagenome pre-processing, assembly, and binning are not always sensitive or fast enough for detection of pathogens in a sample • As sequencing technologies advance, we need new tools to handle output from long- and short-read technologies, as well as single-cell metagenomics approaches	• Easy to install, open-access software with comprehensive documentation detailing best and worst use cases • Defined metrics for critical assessment and validation of existing tools • Software and database versions should be more consistently reported in the literature and preserved for future replication of analyses
Navigating the trade-off between speed and accuracy (Key Conclusion 4) Importance: metagenomic analysis used for pathogen detection and identification are time-sensitive Application example: deciding if a food product should be recalled due to contamination	• Current algorithms vary in speed and accuracy (often sacrificing one for the other) • Large datasets, error-prone heuristics, and coarse resolution of *k*-mer-based methods present challenges	• Better documentation of available tools to help users optimize their software choice based on their available resources • Improvements in sequencing technologies and tools/algorithms to improve both speed and accuracy
Storing and sharing data (Key Conclusion 5) Importance: access to publicly available datasets will help in verification of results and advance of scientific knowledge. Scientists need to be encouraged to move their data out of private silos and into shared databases	• Not all data can be shared because it is important to protect personally identifiable information or intellectual property rights • Lack of sufficient infrastructure or manpower to upload or store datasets at scale	• Defined quality standard to maintain usable, open repositories • Improved ways for secure interrogation of genomic datasets that cannot be openly shared due to privacy regulations

### Sequencing-based assays frequently lack sensitivity

While the biodefense community has benefited from high-throughput sequencing strategies, these methods are not always as sensitive as required. In some cases, culturing is still the most reliable method for detecting pathogens because standard sequencing pipelines are not always available, and achieving required sequencing depths may be cost-prohibitive. Dr. Sarah Allard (UMD SPH) shared her work from CONSERVE (Center of Excellence at the Nexus of Sustainable Water Reuse, Food, and Health), whose mission is to enable the safe use of non-traditional irrigation water sources on food crops [9]. Dr. Allard used both culture-based and sequence-based methods to detect foodborne pathogens in water samples. She concluded that culture-based techniques are currently the most sensitive pathogen detection strategies and that sequencing analysis sensitivity and stringency vary strongly by method.

From a public health perspective, quantification of viable organisms contributing to disease is essential but cannot be achieved with metagenomic analysis alone. Culturing and other approaches are important for gaining insight into the metabolic activity of the microbes in a community [10]. Additionally, researchers must often make a trade-off between the sensitivity of their detection methods and the computational costs of analyzing increasingly deep sequencing datasets. Even partial culturing of select organisms or samples can help shift this trade-off. As commented during a breakout session, “you can’t always sequence your way out of it.”

### Few studies look beyond bacterial pathogens

Shotgun metagenomics and a decrease in the cost of DNA sequencing have enabled researchers to analyze the genetic potential of microorganisms directly from an environmental sample. However, the majority of microbiome and metagenome studies focus only on the prokaryotic component of the community, while few have explored the roles of fungi or viruses in these microbial communities. This is due, in large part, to limitations in resources, laboratory procedures, and in the case of viruses, the lack of a universally distributed marker gene. Additional barriers to mycobiome and virome studies include the ability to obtain sufficient material from low biomass environments, high levels of host contamination, incomplete databases, and a lack of available wet lab protocols and computational analysis pipelines. At the meeting, it was noted that central repositories for shared protocols do exist (e.g., protocols.io [11]), and a concerted effort in viral protocol sharing has been made by the Gordon and Betty Moore Foundation, which funds VERVE Net [12]. Proposed goals to address other barriers included providing financial and/or publication incentives for database curation and maintenance and focusing work on gene function identification. Since the NCBI SRA already contains many metagenomic sequencing datasets, it may be worthwhile to identify novel fungal and viral genomes from existing datasets to optimize data usage, as this approach has been employed in previous studies of environmental viruses [13].

Despite the aforementioned barriers to fungal and viral metagenomics, additional research in this area can significantly contribute to biodefense. One such important topic is the spread of viral pathogens. Invited seminar speaker Dr. Don Milton (UMD SPH) presented his work on the transmission of the influenza virus in college dormitories [14]. The Centers for Disease Control and Prevention (CDC) suggests that human influenza transmission mainly occurs by droplets made when people with flu cough, sneeze, or talk. However, Dr. Milton explained that dueling reviews have disputed the importance of airborne transmission [15–20]. He presented NGS data showing that exhaled breath of symptomatic influenza cases contains infectious virus in fine particles, suggesting that aerosol exposures are likely an important mode of transmission.

### Tracking microbial communities across time and topography

Temporal and biogeographic sequencing studies provide increased resolution of microbial community shifts. In the context of biodefense, this is important for detecting and containing outbreaks. Additionally, these studies provide insight into environmental changes, which may contribute to epidemics by causing shifts in disease vectors and/or spurring human migration to new regions or densely populated urban areas. Several presentations at the meeting shared spatiotemporal microbiome analyses of different environments. Dr. Sean Conlan (NIH, NHGRI) presented his work using metagenomics to study outbreaks of nosocomial infections and identified the transfer of plasmids from patients to the hospital environment [21, 22]. Gherman Uritskiy (JHU) and Dr. Sarah Preheim (JHU) used a combination of marker gene and metagenomics approaches to characterize the changes in environmental microbiomes in response to perturbations. Uritskiy studied halite endoliths from the Atacama Desert in Chile over several years and showed how they were significantly impacted by rainstorms. Dr. Preheim compared a biogeochemical model to microbial communities’ changes in a lake over the spring and summer to reveal the influence of energy availability on microbial population dynamics.

While time series datasets provide valuable information, they are much more difficult to analyze with current statistical methods and models than cross-sectional sampling strategies [23, 24]. Among other reasons, this is because it is difficult to identify the optimal sampling frequency, the compositional nature of microbiome data frequently violates assumptions of statistical methods, and the commonly available software tools are often insufficient for required complex comparisons. Addressing this, Dr. J Gregory Caporaso (NAU) presented QIIME 2 (https://qiime2.org) and shared his team’s QIIME 2 plugin, q2-longitudinal, which incorporates multiple methods for characterizing longitudinal and paired-sample marker gene datasets [25].

### Development and application of metagenomic analysis tools is critical for progress

Computational methods required for metagenomic analyses include taxonomic abundance profiling, taxonomic sequence classification and annotation, functional characterization, and metagenomic assembly. Many of the presentations at the meeting shared new and/or improved tools for different aspects of microbiome studies. Victoria Cepeda (UMD) described how her tool, MetaCompass, uses reference genomes to guide metagenome assembly [26], and Gherman Uritskiy (JHU) presented his pipeline, metaWRAP, for the pre-processing and binning of metagenomes [27]. Furthermore, Brian Ondov (UMD, NIH, NHGRI) shared his implementation of the MinHash containment estimation algorithm to screen metagenomes for the presence of genomes and plasmids [28]. Data visualization is important for accurately interpreting microbiome data analyses, and Dr. Héctor Corrada-Bravo (UMD) demonstrated how to use his lab’s tool, Metaviz [29], for interactive statistical analysis of metagenomes.

Conventional metagenomic analyses often reflect the most abundant elements from a complex sample and cannot detect rare elements with confidence. Dr. Nicholas Bergman (NBACC) shared a more sensitive single-cell metagenomics approach that allows for increased detection of all elements of a community sample. Dr. Bergman’s talk also emphasized the necessity of improving sensitivity, preventing contamination, eliminating biases, and increasing efficiency for sequencing-based techniques.

#### Bioinformatics tools should better match the needs of end users

Many discussions at the meetings focused on how the field can optimize tool utility. It was agreed that scientists should always carefully evaluate the strengths and weakness of available methods, either via existing “bake-off” studies or through the available documentation, to ensure they are using the best tools to address their specific problem. Tool developers should disclose the limits of their methods and advise on the types of data their software is best suited to analyze. Developers should also work towards producing software that is easy to download and install, providing comprehensive documentation for their tools, and ensuring open access for the academic community. As a community, we should encourage that publications list not only cases and data types where methods perform best, but also where they underperform or even fail. Additional studies, like the Critical Assessment of Metagenome Interpretation (CAMI) [30, 31], Microbiome Quality Control project [32], or challenges run under the aegis of PrecisionFDA [33], should be conducted to help characterize the strengths and weaknesses of different approaches and evaluate their impact on data analysis and interpretation.

Some meeting attendees are currently contributing to these goals. Dr. Nathan Olson (UMD, NIST) presented his evaluation of different 16S rRNA marker gene survey bioinformatic pipelines using mixture samples. Additionally, Dr. Daniel Nasko (UMD) characterized how genomic database growth affects study findings, showing that different versions of the RefSeq database strongly influenced species-level taxonomic classifications from metagenomic samples [34]. Because the version of software and databases used can significantly affect the findings, this information should be reported more consistently in the literature. Furthermore, we should consider strategies to preserve previous software and database versions to enable future replication of analyses.

#### Bioinformatics tools must better navigate the trade-off between speed and accuracy

Metagenomic analysis methods vary in the central processing unit (CPU) time, memory, and disk resource usage, and this is not always clearly reported in software publications. Additionally, method scalability relative to size or type of input data also varies considerably. Optimizing speed and accuracy is especially important for biodefense applications. For instance, improvements in NGS analysis allowing for collection and analysis of samples in a clinically relevant time frame can help effectively track hospital outbreaks and prevent the spread of infection [35]. Furthermore, confidence in the accuracy of these analyses is required to execute appropriate plans of action and prevent panic. Recently, findings of *Bacillus* strains on the International Space Station that were genomically similar to pathogenic *Bacillus anthracis* required more detailed characterization to ensure that their presence was not a concern for the health of the crew [36–38]. *B. anthracis* was also initially reported to be found in the NYC subway system, along with *Yersina pestis*, the pathogen responsible for the plague [39]. After public attention prompted further analysis, the authors found no evidence that these organisms were present and found no evidence of pathogenicity [40, 41], again highlighting the importance of careful evaluation and interpretation of results, especially those with severe public health consequences.

Many different strategies for speeding up analyses were discussed at the meeting, including hardware, software, and algorithm choice. Some hardware considerations for the speed of analyses include balancing CPUs with co-processors such as graphics processing units (GPUs) or field-programmable gate arrays (FPGAs), server configuration in terms of the amount of random access memory (RAM), or disk storage type and speed. Programs and algorithms vary in accuracy as well as ease of parallelization. Often a slower yet parallelizable algorithm is preferred to one that is not parallelizable. If a program supports parallelism, consideration should be given to the type of hardware required. For example, some available options include large multicore servers for multithreaded applications, cluster nodes for distribution of compute jobs, or cloud computing solutions. Other strategies might involve analyzing only a subset of the data or using a smaller, application-specific reference database.

Finally, strategies discussed for speeding up time-critical analyses included employing a multi-tiered approach (e.g., a quick first pass followed by more detailed analyses [42]) and considering the suitability of various sequencing platforms for certain applications. Interventions or optimizations were discussed with regard to their impact on analysis accuracy and interpretation of results. Preferred solutions are the ones that provide both the desired speed and accuracy, though more often than not there is a trade-off between the two. The optimal balance also depends on the use case. Assessment and validation methods are required to characterize a method’s speed and accuracy. It will be up to the subject matter experts to determine the desired accuracy level for each case and the extent to which they can sacrifice accuracy for speed.

### Data needs to be moved out of private silos and into public repositories

Data sharing is continually a challenge that gets raised within the biological community, especially as DNA/RNA sequencing becomes more ubiquitous and tangible outside of core facilities [43]. This challenge is prevalent across multiple scientific disciplines and was recently highlighted by the National Research Council as a priority for microbial forensics [44]. There are numerous reasons data are not being shared, including the need to protect personally identifiable information or intellectual property rights prior to publication and the lack of sufficient infrastructure or manpower to upload at scale. However, leveraging this diversity and breadth of data will be important for an effective biodefense capacity, as well as other bioscience applications like healthcare, pharmaceuticals, agriculture, and industry. In order to incentivize data sharing, we need to evaluate and improve publicly available resources for storing and processing data.

Inherent altruism or obligation to share data should be met with as little friction as possible, and we need to incentivize openness. One incentive is academic credit through authorship on publications, though this will require combined efforts of researchers, journal editors, and funding agencies to better define what contributions constitute data authorship and what responsibilities data authors have [45, 46]. Another potential incentive is the availability of free software for data analysis and meeting participants debated the desirability and sustainability of service-based options (e.g., MG-RAST [47]) compared to user-installable software options (e.g., QIIME [48], mothur [49]). At the meeting, Dr. Nur A. Hasan (CosmosID, Inc.) highlighted the cloud-based metagenome tools and databases his company has to offer. There are also strong movements towards software sharing, such as the Astrophysics Source Code Library [50] and the Materials Resource Registry at NIST [51].

It is expected that some quality standard is needed to maintain usable, open repositories. Where that standard is set can affect how much data is shared. For example, a high bar may ensure high-quality sequences and comprehensive metadata but minimize sharing, while a lower quality bar will more likely move data out of silos. The solution may be a combination of repositories with varying standards or a single repository which allows for varying degrees of annotation completeness and allows the user to modify searches based on that feature. It is important to note that a single repository may be difficult to reliably curate and manage at scale. Another option is distributed but federated systems, like used by the US Virtual Astronomical Observatory [52]. Groups like the Genomic Standards Consortium [53, 54] are working towards improving data quality by supporting projects such as Minimum Information about any Sequence (MIxS) [55], which establishes standards for describing genomic data and provides checklists to help with annotation. We need to build a community consensus on how much metadata is required to make reporting less onerous for data providers but ensure data usability by others in the field.

Incentivizing open data sharing should not be the only solution, as some sensitive data cannot be openly shared due to privacy regulations (e.g., human genomes and Health Insurance Portability and Accountability Act regulations). Other sectors, such as the financial industry, have long been working on solutions to enable storage, transit, and operations of protected data. These solutions include software-based approaches (e.g., homomorphic encryption, Yao’s protocol, secure fault-tolerant protocols, oblivious transfer) and hardware-based approaches (e.g., AES full disk encryption for data storage, Intel® Software Guard Extension for secure operations). Dr. Stephanie Rogers presented the GEMStone 2.0 project from B. Next, an IQT Lab, called SIG-DB, which explores homomorphic encryption and Intel Software Guard Extension (SGX) to securely search genomic databases [56]. Early results of applying these solutions to biological data are promising and should be explored more fully.

## Conclusions

Overall, this meeting successfully brought together scientists from academia, government, and industry to present their research and discuss how high-throughput genomics methods have stimulated interest and progress in biodefense and pathogen detection. Notably, meeting participants used NGS tools to identify the transfer of microbes from patients to their hospital environments, track the transmission of influenza in a community living space, study environmental shifts over time, and evaluate the safety of using non-traditional water sources on food crops. These studies, and others, have been partly driven by cheaper, more reliable sequencing technologies and improvements in computational analysis tools. Open-source software for sequence processing and quality control, taxonomic annotation, metagenomic assembly, and binning, and data visualization have been essential for growth. Continued development of these resources will result in significant scientific advances.

Despite this progress, there are several limitations to using NGS approaches for biodefense problems. First and foremost, sequencing methods are unable to accurately quantify viable organisms from metagenomic samples, which is essential for identifying potential threats to public health. Beyond that, applications for which NGS approaches are well-suited still present many challenges. Although sequencing costs are steadily declining, it remains expensive to process, computationally analyze, and store the increasingly large datasets that are generated. Confident detection of infectious, but potentially rare pathogens in a community often requires very deep sequencing, and scientists must make the appropriate speed, cost, and accuracy trade-offs to best answer their research questions. In many cases, sequencing experiments may need to be complemented with culturing, enrichment, or other targeted approaches. Because of these limitations, and others, researchers must be extremely careful when interpreting data to identify biothreats; reporting false positives without critical validation can have significant fiscal and public health consequences. Developing the capacity to identify not only when a potential pathogen is present but also at what levels it is actively contributing to an infectious disease will greatly improve our response to biothreats. Another area that requires further investigation is the detection of antimicrobial resistance. While only briefly highlighted in the meeting talks about influenza and nosocomial tracing, antimicrobial resistance poses a significant threat to public health and biodefense. Current metagenomic sequencing methods allow us to identify antimicrobial resistance genes from different environments; however, these techniques cannot determine whether these genes are actively being expressed and are currently not practical for wide-spread adoption in clinical settings [57].

To date, few microbiome studies have focused on viral and fungal/eukaryotic organisms, despite their potentially important community interactions and roles in pathogenesis. In order to generate relevant virome and mycobiome datasets, we must improve sample processing techniques and dedicate resources to effectively curate and maintain publicly available databases. We also need to develop advanced statistical toolkits for analyzing longitudinal studies. In general, tool developers should focus on creating user-friendly, adaptable resources, with comprehensive documentation and clear descriptions of default settings and optional parameters. These tools must be critically evaluated for their appropriate use cases; however, when looking for emerging threats, it will be necessary to develop validation approaches that do not require the use of gold standards.

In order to encourage additional growth, the greater scientific community should invest in expanding and enforcing clear standards for genomic datasets. If set appropriately, these standards will help incentivize data sharing and improve the quality and usability of public repositories. Additional focus should be on strengthening best practices and solutions for handling sensitive datasets that are subject to privacy regulations. Moving forward, active conversations between researchers and policymakers will be essential to expand and implement these ideas in biodefense.

## Additional file

Additional file 1: **Table S1.** Outline of oral presentations at the January 2018 M^3^ Meeting. **Table S2.** Outline of interactive breakout sessions at the January 2018 M^3^ Meeting. (DOCX 20 kb)

## Abbreviations

CBCB: Center for Bioinformatics and Computational Biology
CONSERVE: Center of Excellence at the Nexus of Sustainable Water Reuse, Food, and Health
CPU: Central processing unit
FPGA: Field-programmable gate array
GPU: Graphics processing unit
IQT: In-Q-Tel, Inc.
JHU: Johns Hopkins University
M^3^: Mid-Atlantic Microbiome Meet-up
NAU: Northern Arizona University
NBACC: National Biodefense Analysis and Countermeasures Center
NGS: Next-generation sequencing
NHGRI: National Human Genome Research Institute
NIH: National Institutes of Health
NIST: National Institute of Standards and Technology
RAM: Random access memory
SPH: School of Public Health
UMD: University of Maryland
