# Supplementary material for: Current progress and future opportunities in applications of bioinformatics for biodefense and pathogen detection: report from the Winter Mid-Atlantic Microbiome Meet-up, College Park, MD, January 10, 2018
Source: Microbiome. 2018 Nov 5;6:197. doi: 10.1186/s40168-018-0582-5 (PMC6219074; doi:10.1186/s40168-018-0582-5)
Supplement: Supplementary file 1 — Table S1. Outline of oral presentations at the January 2018 M3 Meeting. Table S2. Outline of interactive breakout sessions at the January 2018 M3 Meeting. (DOCX 20 kb) [file 40168_2018_582_MOESM1_ESM.docx]

**Table S1** Outline of oral presentations at the January 2018 M^3^ Meeting

|  | **Speaker** | **Title** |
| --- | --- | --- |
| **Keynote** | Tara O'Toole  In-Q-Tel, Inc. | Bioterror, and Biodefense, 2018 |
| **Invited Seminar** | Don Milton  UMD SPH | College Dorms as a Laboratory for Studying Respiratory Infection |
| **Data-driven session**  Chair:  Stephanie Rogers  B.Next  (In-Q-Tel, Inc.) | Stephanie Rogers  B.Next (In-Q-Tel, Inc.) | Bridging technology, venture, and national security |
|  | Daniel Nasko  UMD CBCB | Tragedy of the commons: RefSeq database growth influences the accuracy and sensitivity of species identification from metagenomic samples |
|  | Sarah Allard  UMD SPH | Comparison of sequencing and culture-based methods for the detection of foodborne pathogens in non-traditional irrigation water in the Mid-Atlantic United States: A CONSERVE study |
|  | Sean Conlan  NHGRI, NIH | Tracking Antibiotic Resistance Across Space and Time |
|  | Greg Caporaso  NAU, NCI, NIH | Longitudinal analysis of microbiomes |
| **Methods-driven session**  Chair:  Nicholas Bergman  NBACC | Nicholas Bergman  NBACC | Metagenomics in bioforensics |
|  | Brian Ondov  UMD, NHGRI, NIH | Mash Screen: Fast sequence containment estimation using MinHash |
|  | Nathan D. Olson  UMD CBCB and  NIST | A Sample Mixture Experiment to Assess 16S rRNA Metagenomic Methods |
|  | Victoria Cepeda  UMD CBCB | MetaCompass: Reference-guided Assembly of Metagenomes |
|  | Héctor Corrada-Bravo  UMD CBCB | Metaviz: Interactive Statistical and Visual Analysis of Human Microbiome Project Data |
| **Ecology-driven session**  Chair:  Jocelyne DiRuggerio  JHU | Gherman Uritskiy  JHU | Dynamic Response of Atacama Desert Extremophiles to Weather Perturbations |
|  | Sarah Preheim  JHU | Frequency and impact of transitions between microbial populations mediating biogeochemical cycling in a freshwater lake |
|  | Nur A. Hasan  CosmosID | Cloud based bioinformatics platform for cross-disciplinary microbiome research |

**Table S2.** Outline of interactive break-out sessions at the January 2018 M^3^ Meeting

| **Break-out Session** | **Chairs** | **Topics** |
| --- | --- | --- |
| Viral and fungal pathogens: off the beaten path | Jacquelyn Meisel  UMD CBCB  and Daniel Nasko  UMD CBCB | - Barriers to studying viruses and fungi and actionable goals to address - Detection of viral and fungal pathogens |
| Crowdsourcing biodefense: data sharing, data standards, data  security, and data visualization | Todd Treangen  UMD CBCB  and Brian Ondov  UMD, NHGRI, NIH | - Homomorphic encryption techniques for secure computations - Data visualization as incentive for data sharing - Data standards: raising the bar to ensure quality vs lowering the bar to bring data out of silos - Cloud based data sharing: practical or impractical? |
| Need for speed: navigating the trade‑off between analysis  accuracy and speed | Adam Bazinet  NBACC  and Nathan D. Olson  UMD CBCB and NIST | - Evaluation of time-sensitive metagenomic analyses - Strategies for speeding up such analyses without compromising accuracy |
